# Mobile robotics platform for strawberry temporal–spatial yield monitoring within precision indoor farming systems

**DOI:** 10.3389/fpls.2023.1162435

**Published:** 2023-04-25

**Authors:** Guoqiang Ren, Hangyu Wu, Anbo Bao, Tao Lin, Kuan-Chong Ting, Yibin Ying

**Affiliations:** ^1^ College of Biosystems Engineering and Food Science, Zhejiang University, Hangzhou, Zhejiang, China; ^2^ Zhejiang University-University of Illinois Urbana-Champaign Institute (ZJU-UIUC), International Campus, Zhejiang University, Haining, Zhejiang, China; ^3^ Key Laboratory of Intelligent Equipment and Robotics for Agriculture of Zhejiang Province, Hangzhou, China; ^4^ College of Control Science and Engineering, Zhejiang University, Hangzhou, Zhejiang, China; ^5^ Department of Automation, Shanghai Jiao Tong University, Shanghai, China; ^6^ Department of Agricultural and Biological Engineering, University of Illinois at Urbana-Champaign, Urbana, IL, United States

**Keywords:** mobile robotics platform, indoor vertical farming systems, GPS-denied navigation, temporal–spatial data collection, yield monitoring

## Abstract

Plant phenotyping and production management are emerging fields to facilitate Genetics, Environment, & Management (GEM) research and provide production guidance. Precision indoor farming systems (PIFS), vertical farms with artificial light (aka plant factories) in particular, have long been suitable production scenes due to the advantages of efficient land utilization and year-round cultivation. In this study, a mobile robotics platform (MRP) within a commercial plant factory has been developed to dynamically understand plant growth and provide data support for growth model construction and production management by periodical monitoring of individual strawberry plants and fruit. Yield monitoring, where yield = the total number of ripe strawberry fruit detected, is a critical task to provide information on plant phenotyping. The MRP consists of an autonomous mobile robot (AMR) and a multilayer perception robot (MPR), i.e., MRP = the MPR installed on top of the AMR. The AMR is capable of traveling along the aisles between plant growing rows. The MPR consists of a data acquisition module that can be raised to the height of any plant growing tier of each row by a lifting module. Adding AprilTag observations (captured by a monocular camera) into the inertial navigation system to form an ATI navigation system has enhanced the MRP navigation within the repetitive and narrow physical structure of a plant factory to capture and correlate the growth and position information of each individual strawberry plant. The MRP performed robustly at various traveling speeds with a positioning accuracy of 13.0 mm. The temporal–spatial yield monitoring within a whole plant factory can be achieved to guide farmers to harvest strawberries on schedule through the MRP’s periodical inspection. The yield monitoring performance was found to have an error rate of 6.26% when the plants were inspected at a constant MRP traveling speed of 0.2 m/s. The MRP’s functions are expected to be transferable and expandable to other crop production monitoring and cultural tasks.

## 1 Introduction

Strawberries (Fragaria × ananassa) are favored by consumers due to their rich nutrition and distinctive flavor. Precision indoor farming systems (PIFS), vertical farms with artificial light (aka plant factories) in particular, have long been suitable plant production scenes due to the advantages of efficient land utilization and year-round cultivation. In recent years, some companies, including Bowery Farming, Oishii Farm, and 4D Bios, successfully cultivated strawberries in plant factories. Farmers and researchers need to understand how plants grow and provide what plants need to increase fruit yield and quality. Plant phenotyping, an emerging science that describes the formation process of the functional plant body (phenotype) under the influence of dynamic interaction between the genotypic differences (genotype) and the corresponding environmental conditions (Walter et al., 2015), can provide valuable information for crop genetic selection and production management. People usually go to fields or laboratories to manually obtain plant phenotypic data. Such practices are highly labor-intensive, time-consuming, non-robust, and sometimes destructive and, therefore, may be limited by experimental scale, collection accuracy, and human subjective differences (Bao et al., 2019). A field-based, large-scale, and high-throughput plant phenotyping approach to overcome the bottleneck of manual operation is urgently needed (Araus et al., 2018).

Internet of Things (IoT) devices, which focus on collecting environmental data, are prevalent within PIFS as the monitoring system. Experience-oriented growth regulation decision-making can be built using environmental data by production managers. However, the decision-making process based on experience is indirect and delayed. The plant phenotypic data should be added to form a closed-loop decision-making pipeline. Considering fine-grained data collection is positively correlated with the number of camera sensors, the coverage and accuracy of data acquired by traditional IoT systems cannot be readily achieved within reasonable budgets. Mobile robots equipped with multiple sensors (the concept of quasi-IoT) present a great potential to acquire desired phenotyping data automatically. In the past few years, reported examples of phenotyping robots, emphasizing mobility-enabled field trials, have been increasing (Mueller-Sim et al., 2017; Shafiekhani et al., 2017; Higuti et al., 2019). However, there has been limited published work on mobile robots that have the capability of autonomously capturing phenotypic data within PIFS. We aimed to develop a mobile robotics platform (MRP) with the capabilities of periodical monitoring of individual strawberry plants and fruit within the entirety of a commercial plant factory. Fine-grained plant growth data captured by the MRP can provide production guidance and facilitate integrated GEM research.

An MRP applied in agricultural scenarios should have two primary capabilities: providing navigation for multiple-location data acquisition and data-driven decision support. Navigation in indoor scenarios is challenging due to the lack of GPS. As an alternative approach to GPS used in indoor scenarios, ultra-wideband (UWB) is high-precision but high-cost (Flueratoru et al., 2022). The stability of the navigation is closely related to the strength of signals that suffer from occlusion and attenuation errors under plant growing structures. Furthermore, UWB provides relatively static information that cannot detect unexpected obstacles. Light Detection and Ranging (LiDAR) sensors have been widely used in agricultural navigation that can actively acquire accurate depth information with an extensive detection range and a low sensitivity to lighting changes compared to other sensors (Debeunne and Vivet, 2020). A random sample consensus (RANSAC) algorithm was applied to discern maize rows fast and robustly while navigating in a well-structured greenhouse (Reiser et al., 2016). However, in complex environments like plant factories with repetitive shelves and narrow aisles, LiDAR can only obtain a limited number of signals representing the presence of objects. There is no semantic information for effectively completing the scene restoration. In contrast, visual navigation is limited by the low accuracy in depth estimation and the weak robustness against lighting changes (Zhang et al., 2012). A robot cannot safely and robustly navigate within plant factories using only one sensor as the single perception source. Multi-sensor fusion approaches, which can significantly improve the fault tolerance of a system while increasing the system’s redundancy to increase the accuracy of object localization, have been proven to show great potential to solve navigation problems in complex scenes like urban traffic (Urmson et al., 2008). In consideration of a GPS-denied environment like PIFS, simultaneous localization and mapping (SLAM) technology can be a feasible navigation approach (Chen et al., 2020). The state-of-the-art LiDAR-SLAM Cartographer (Hess et al., 2016) and visual–inertial system (VINS) (Qin et al., 2018) are all open-source tools in the ROS (Robot Operating System) community. These algorithms, which can be easily implemented on a mobile robot, can potentially address navigation challenges. However, SLAM has some limitations, such as computational cost and lack of feature extraction ability; therefore, it is not directly applicable to this research. In this study, we report our research on a novel approach of fusing wheel odometry, inertial measurement unit (IMU), and AprilTag observations (captured by a monocular camera) to achieve accurate navigation within repetitive and narrow passages of PIFS.

Providing data-driven decision support based on the plant growth information is the other critical capability of the MRP. There exist some common decision-making pipelines in both academia and industry, including ripeness detection (Talha et al., 2021), diseases and pest identification (Lee et al., 2022), and fruit counting (Kirk et al., 2021). Image data captured by various perception systems have been widely used to achieve the above purpose (Gongal et al., 2015). In recent years, AlexNet brought about a renewed understanding of deep CNN and evolved into the foundation of contemporary computer vision (Krizhevsky et al., 2012). The powerful end-to-end learning makes the decisions possible, especially in the detection-based task from static images (Zhou et al., 2020; Perez-Borrero et al., 2021). The computing power of MRP limits the development of efficient CNN architectures as the neural network deepens (Zhang et al., 2018). Both occlusions from neighboring fruit and foliage and illumination changes could cause variations in fruit appearance (Chen et al., 2017). Compared to tasks, like ripeness and disease detection, counting from videos is challenging due to bias in fruit localization and tracking errors originating from occlusions and illumination changes (Liu et al., 2018b). Some traditional algorithms, including Optical Flow, Hungarian algorithm, and Kalman Filters, were used to track multiple fruits among sequential video frames. Liu et al. combined fruit segmentation and Structure from Motion (SfM) pipelines for counting apples and oranges grown on trees. The extra introduction of relative size distribution estimation and 3D localization could eliminate parts of double-counted fruits to further enhance the counting accuracy. Strawberry fruit is of small sizes and has complex ripe stages and dense growth scenes, which bring real challenges to the detection and tracking process.

This paper reports the current state of development and testing of the MRP’s abilities of periodical monitoring of individual strawberry plants and fruit within a commercial plant factory. The challenges of navigation within narrow and repetitive indoor environments for temporal–spatial plant data acquisition and accurate yield monitoring for production management and harvesting scheduling in the MRP’s periodical inspection operations need to be taken into consideration. In summary, the objectives of our research are as follows:

1. To develop the software and hardware of an MRP, consisting of an autonomous mobile robot (AMR) and a multilayer perception robot (MPR), which can capture temporal–spatial phenotypic data within a whole strawberry factory.
2. To achieve accurate navigation within the repetitive and narrow structural environments of a PIFS through an AprilTag and inertial navigation (ATI navigation) algorithm.
3. To evaluate the performance of strawberry yield monitoring through a novel pipeline that combines keyframes extraction, fruit detection, and postprocessing technologies.

## 2 Mobile robotics platform

**Figure 1:**
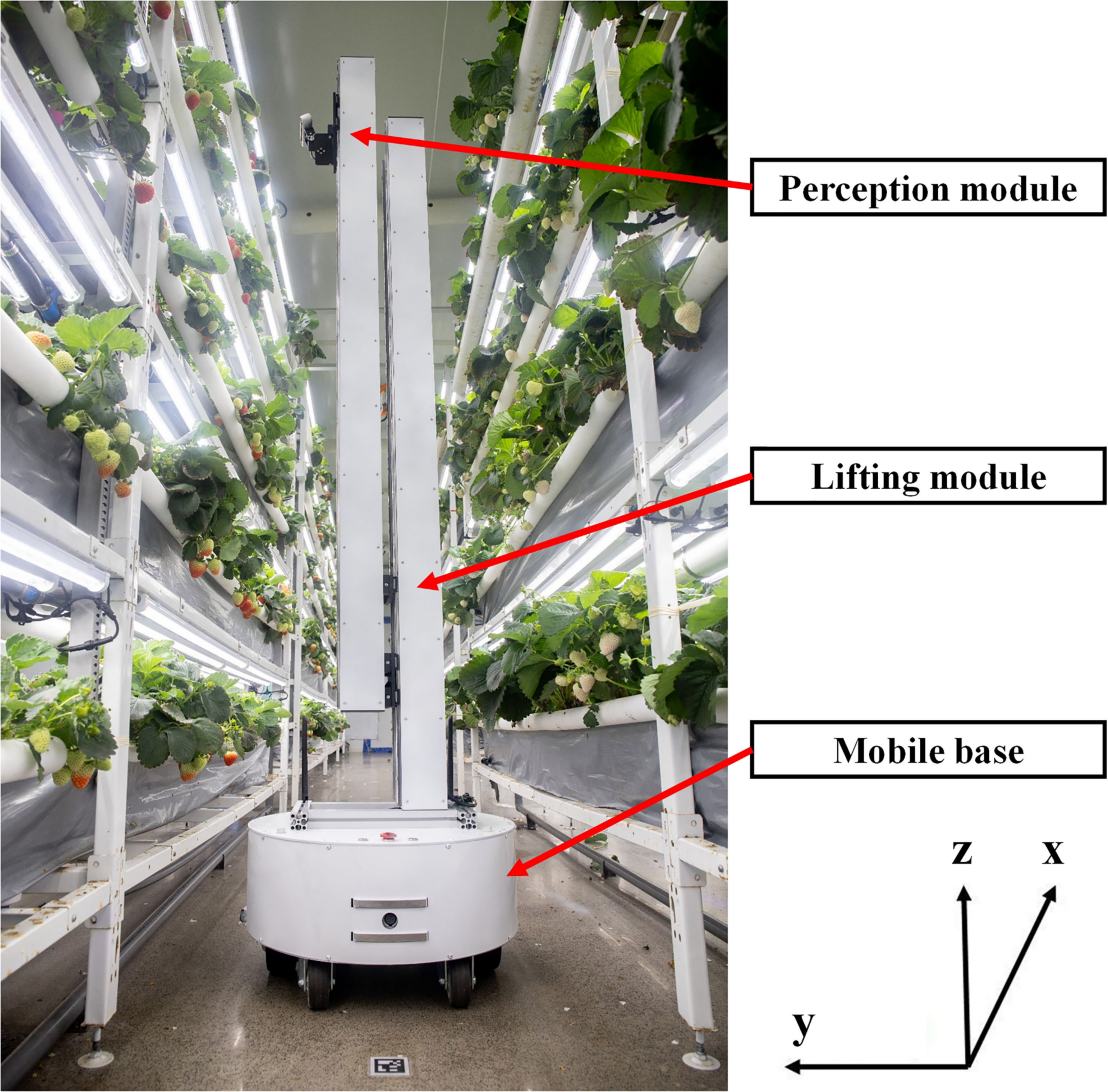
Hardware of the mobile robotics platform (MRP).

In this study, an MRP to operate within a PIFS with multiple plant growing tiers has been developed to dynamically monitor plant growth and provide data for supporting crop growth model construction and production management. The modularly designed MRP (**Figure 1**) consists of an AMR, i.e., the mobile base, and an MPR, i.e., the lifting module + perception module, where MRP = MPR installed on top of AMR. The AMR is capable of traveling along the aisles between plant growing rows (i.e., *x* direction) with high positioning accuracy (PA) and robust navigation capability. The MPR has a perception module (for data acquisition) that can be raised by a lifting module to reach the heights (*z* direction) of all plant growing tiers of every row within the PIFS. The assembly of the AMR and MPR can perform automatic acquisition, storage, and transmission of phenotypic data of all individual plants within the entirety of a plant factory. Furthermore, multiple fault detection measures were designed and installed in the MRP. The MRP has been operating in a commercial strawberry production plant factory since July 2022, and has been working as expected so far.

The AMR is a differential drive mobile robot with two 165-mm hub motors, which has the ability to turn on the spot. The cylinder shape mobile base has a diameter of 500 mm and a height of 240 mm, which can travel at a maximum speed of 1.5 m/s through an aisle (with a minimum width of 600 mm) within a plant factory. An Intel^®^ Core™ i5-8265U/1.6 GHz industrial computer is mounted inside the robot to run all navigation, data acquisition, and data transmission programs. The speed control commands from the industrial computer can be received by a low-level control board to drive the AMR to move. Wheel encoders, an IMU (US$40) mounted inside the mobile base, and a downward viewing monocular camera (US$25) to detect AprilTags on the floor are integrated to realize accurate localizations within PIFS, and a 2D LiDAR is used to detect obstacles. An emergency button is directly connected to the low-level control board to stop the motors when necessary.

The MPR is for use to perform data acquisition. The perception module of the MPR is an Intel^®^ RealSense™ D435i depth camera (Intel Corporation, California, USA) mounted on a servo motor that provides the camera with the pitch motion to capture multiple images from various camera angles. The perception module can be raised to 2.8 m, the height of the top tier of each plant growing row, by the lifting module. The phenotypic data of each plant within a strawberry PIFS can be collected by the MRP’s periodical inspection of the entire facility. Data of all plants on one of the five tiers were collected on one inspection route. The data of plants and the MRP’s motion can be recorded in the rosbag format at a unified timestamp, which facilitates the data analysis and decision support processes. During the experiments on data acquisition, the MRP traveled at the speeds of 0.2, 0.3, and 0.4 m/s along the aisle between plant growing rows. The distance between the center of the MRP and the sides of the plant growing rows was kept at approximately 410 mm. The resolution of the RealSense camera was set to 1,280 × 720 at 30 frames per second (FPS). The camera was set to be parallel to the side of a plant growing row by a servo motor and at the same height as the fruit by the lifting module. The same procedure was conducted to ensure the success of data acquisition on each tier.

## 3 Methods

This section presents two basic capabilities of the MRP: navigation for multiple-location data acquisition and strawberry yield monitoring.

### 3.1 Navigation

**Figure 2:**
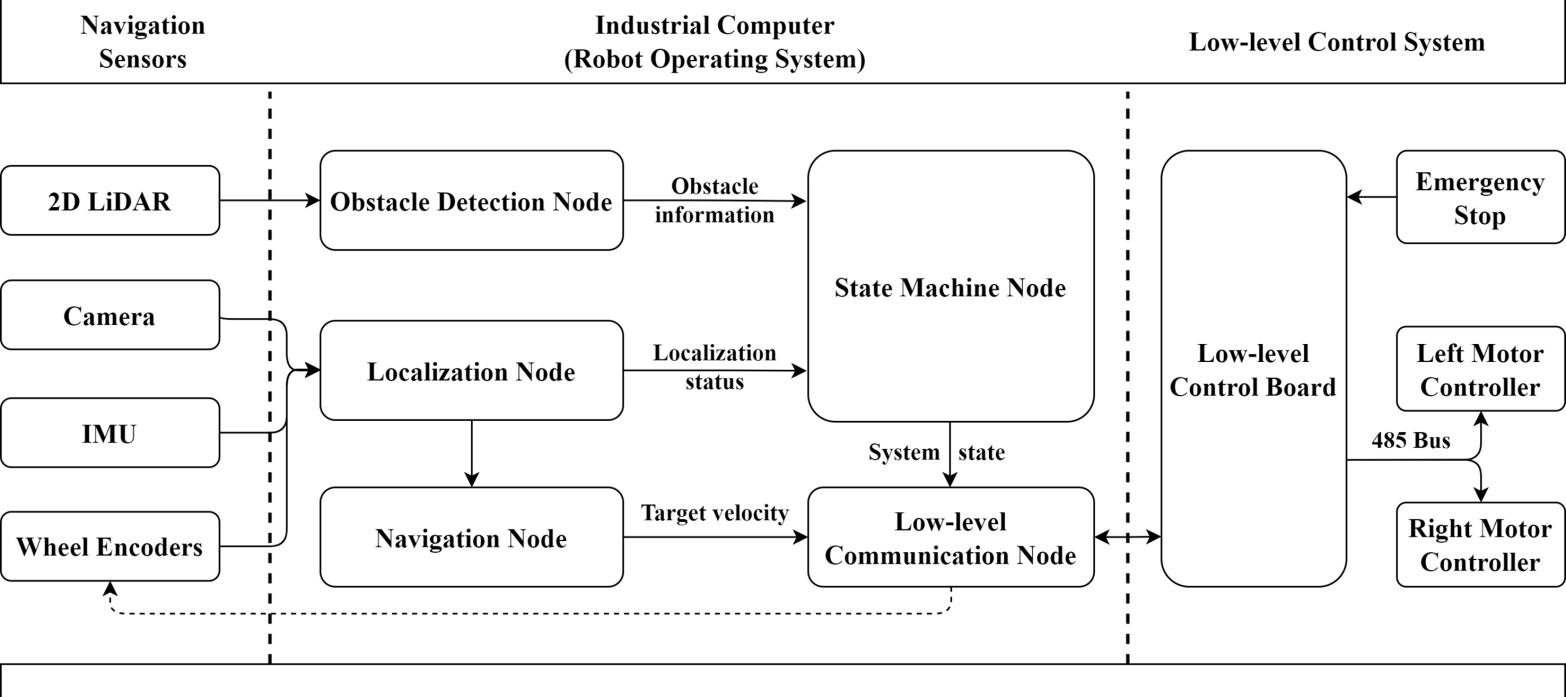
Navigation system architecture.

The navigation system installed in the AMR included navigation sensors, an industrial computer, and a low-level control system (**Figure 2**). The ROS was implemented in the industrial computer to collect data and conduct the navigation pipeline. There were five ROS nodes in the navigation pipeline, including an obstacle detection node, a localization node, a navigation node, a state machine node, and a low-level communication node. The real-time poses (position and heading) of MRP were calculated from the camera, IMU, and wheel encoders, through the localization node. The poses were received by the navigation node to conduct the global path planning and local path tracking, which, in turn, generated the target angular velocity and linear velocity of the MRP at a frequency of 50 Hz. The obstacle information captured by a 2D LiDAR from the obstacle detection node and the localization state (success or failure) from the localization node were sent to the state machine node. The updated state of the system from the state machine node and the target velocity from the navigation node were transmitted to the low-level communication node, which then calculated the target speed of the two motors and sent them to the low-level control board through serial communication.

An ATI navigation algorithm was developed to address the challenges of accurate navigation within the repetitive and narrow structural environments of a PIFS. The ATI navigation algorithm consists of four parts: mapping, localization, planning, and control.

**Figure 3:**
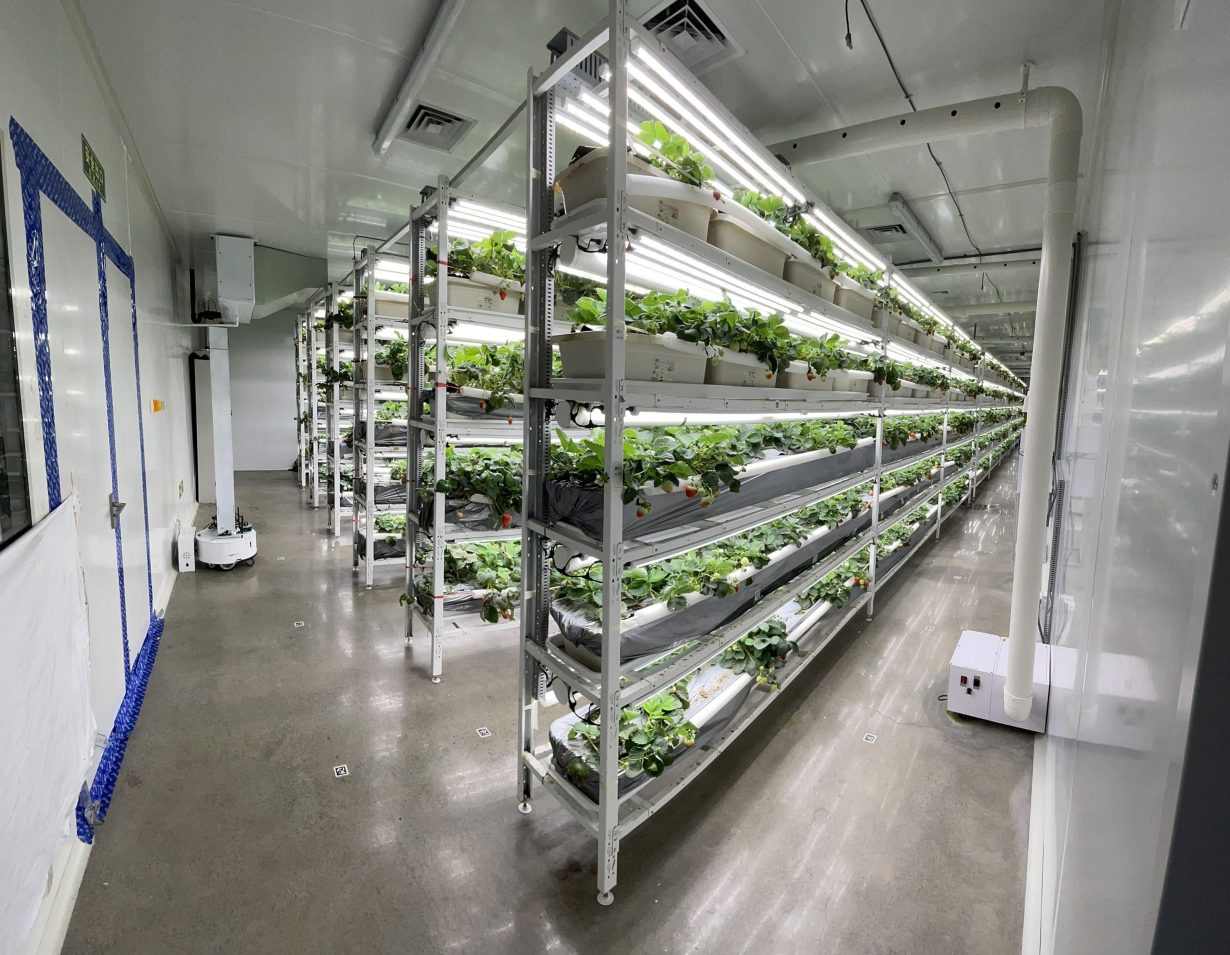
The MRP is being charged in the commercial strawberry factory.

The purpose of mapping in this study was to chart the moving route of MRP. The research was carried out at a commercial strawberry factory (4D Bios Inc., Hangzhou, China). A total of 45 AprilTags (Olson, 2011) of 40 × 40 mm in size were pasted on the grounds of both sides of each plant growing row. An 875 Prolaser^®^ (KAPRO TOOLS LTD., Jiangsu Province, China) was used to ensure that all tags were on a designated straight line. The distance between the two neighboring tags was approximately 1.3 m. When collecting data for developing the ground map of a production facility, the MRP was first moved to Tag 0, which is the location of the charging pile (**Figure 3**). The MRP was controlled by a joystick to pass above the tags in order while simultaneously recording the data of the monocular camera, IMU, and wheel encoders. The mapping dataset was built after MRP had traveled along all the tags and returned to Tag 0.

The tag ID and the homogeneous transform of the tag relative to the monocular camera mounted on the MRP were both calculated by the AprilTag detection algorithm (Wang and Olson, 2016). The wheel encoders and IMU were fused to calculate the trajectory of the MRP using Equation 1.


(1) $$\{\begin{array}{c} \theta_{k+1}=\theta_{k}+\text{Δ}\theta_{imu}\\ x_{k+1}=x_{k}+\left(\text{Δ}s_{l}+\text{Δ}s_{r}\right)\cos\left(\theta_{k}\right)/2\\ y_{k+1}=y_{k}+\left(\text{Δ}s_{l}+\text{Δ}s_{r}\right)\sin\left(\theta_{k}\right)/2 \end{array}$$

where $\text{Δ}\theta_{imu}$ is the heading variation of IMU between timestamps of k and k+1 . $\text{Δ}s_{l}$ and $\text{Δ}s_{r}$ represent the motions of the left and right wheel obtained by optical encoder during two timestamps, respectively.

The tag IDs were further used to conduct the loop closing optimization through the pose graph optimization (PGO) algorithm. The vertices were represented by processed global poses of the tags, and the edges were denoted by relative pose changes of the odometer while MRP accessed two neighboring tags. We cast this as a nonlinear least squares problem


$$\underset{x}{\arg\min}\frac{1}{2}\sum_{ij}e_{ij}^{T}\Omega_{ij}e_{ij}\;$$

where the state of the tag is denoted by a 2D coordinate vector and a heading angle, x={p, θ} . The information matrix $\Omega_{ij}$ is used to assign weights to different errors. The error $e_{ij}$ between the expected observation and the real observation from Tag i and Tag j, can be calculated by Equation 2.


(2) $$e_{ij}=\left(\begin{array}{c} R_{i}^{T}\left(p_{j}-p_{i}\right)-{\hat{p}}_{ij}\\ \theta_{j}-\theta_{i}-{\hat{\theta }}_{ij}\; \end{array}\right)$$

$R_{i}$ is the rotation matrix corresponding to the heading angle in $x_{i}$ . ${\hat{p}}_{ij}$ and ${\hat{\theta }}_{ij}$ represent the relative pose changes of edges. Levenberg–Marquardt (L-M) algorithm was used to optimize the poses of all tags and generate the map. The accurate poses of the tags could be obtained in the process of mapping.

Based on whether one of the AprilTags was detected at the current timestamp, the estimations of localization could be divided into two situations. When the tag was correctly detected by the monocular camera, the global pose of the MRP at this timestamp could be calculated by the global pose of the tag in the existing map and the pose transform of the tag relative to the MRP. Otherwise, the detection result of the last tag in the existing map and the odometry changes from the timestamp when the last tag was detected to the current timestamp were used to estimate the global pose of the MRP.

In path planning, based on the destination, on the mapped route, entered by a human operator, a trajectory composed of a sequential set of locations could be generated by MRP’s global path planner as the waypoints. Based on whether the destination is a tagged position, global path planning can be divided into two cases. If the destination is the position of one of the tags on the undirected map, the shortest path can be obtained through the breadth-first search (BFS) algorithm. If not, a virtual tag representing the destination will be temporarily inserted between two adjacent tags on the undirected map. The optimal path could be calculated by the BFS algorithm performed on the newly constructed undirected map.

After obtaining the global path, the MRP can be navigated through a series of local paths at the angular and linear velocities issued by the low-level control board (**Figure 2**). For a straight global path consisting of more than or equal to three tags, the local path target position is set to $Tag_{i+2}$ with MRP passing $Tag_{i}$ , which will keep the velocity of the MRP along the planned route stable. Angular velocity is calculated by the anti-windup pi controller to adjust the heading toward the target position. The linear velocity is calculated by a proportional controller to prevent system overshoot. The target speed of the left and right motors will be further obtained according to the differential motion model.

### 3.2 Yield monitoring

The growth condition of strawberries on each tier of the plant growing rows could be recorded in a video format after the inspection by the MRP. In this study, we have developed a strawberry yield monitoring method. The counting-from-video method consisted of two phases: detection and counting of ripe fruit.

#### 3.2.1 Fruit detection

Ripeness detection is the first step in the yield monitoring pipeline. Considering that the detection task has high requirements for speed and accuracy, the single-stage detector YOLOv5 is chosen to detect the ripe strawberry (Jocher et al., 2022). The framework of the detector can be divided into four parts: Input with mosaic data augmentation, CSPDarknet53 (Bochkovskiy et al., 2020) as Backbone, Neck applying Feature Pyramid Network (FPN) (Lin et al., 2017) and Path Aggregation Network (PAN) (Liu et al., 2018a), and Prediction using GIoU loss (Rezatofighi et al., 2019). The framework extracts and aggregates semantically and spatially strong features more efficiently. More efficient representation improves the performance of multi-scale object recognition. Various variants have been generated by adjusting the depth and width of the network. YOLOv5l6 was used in this research, with an inference time of 15.1 ms running on an NVIDIA^®^ V100 Tensor Core GPU.

#### 3.2.2 Fruit counting

**Figure 4:**
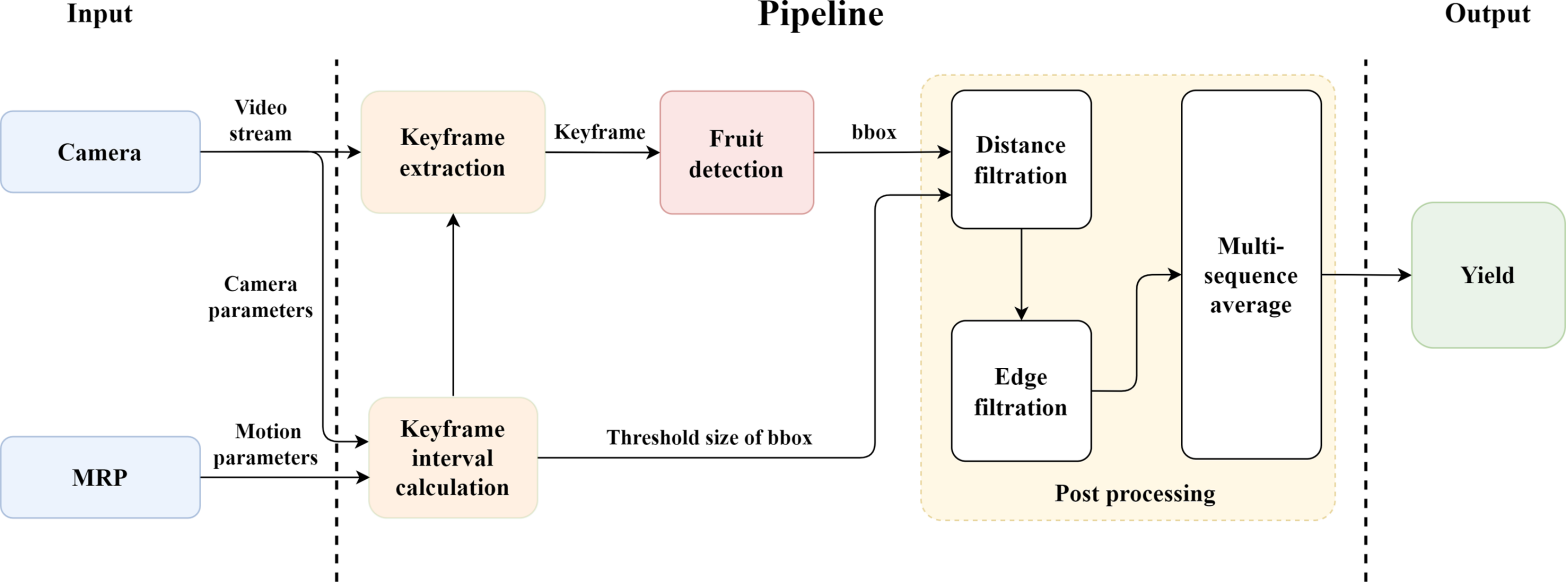
The overall yield monitoring pipeline.

A fruit counting pipeline was presented to count ripe strawberries on video, including keyframe extraction, fruit detection, and postprocessing (**Figure 4**).

##### 3.2.2.1 Keyframe extraction

**Figure 5:**
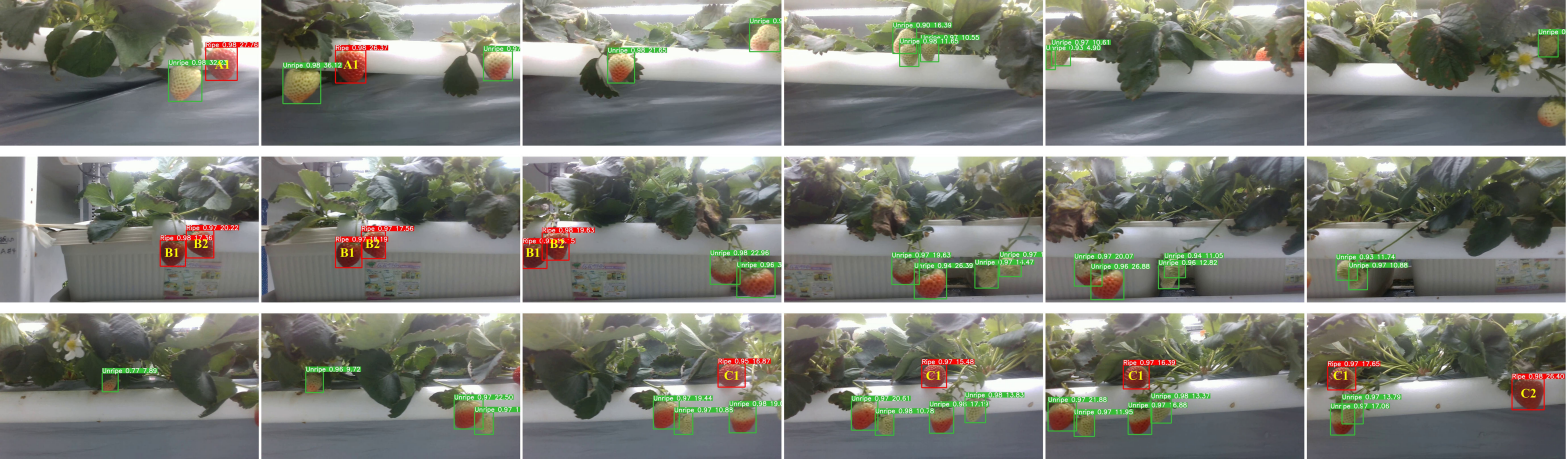
Example series of keyframes at various values of r : r=2 in the upper row, r=3 in the middle row, and r=4 in the bottom row.

Considering that any individual strawberry fruit could appear in multiple frames of the video captured, the number of times a fruit might be counted was not fixed. Therefore, fruit detection results could not be directly accumulated to obtain the counting results. The concept of keyframe extraction was applied to fix the number of times of repetitive counting, r . The pixel distance of two neighboring keyframes in the pixel coordinate system, $d_{p}$ , was calculated by Equation 3.


(3) $$d_{p}=\frac{w}{r}$$

where w was the image width. All strawberries in the video were required to appear at least twice in all extracted frames; therefore, r was greater than or equal to 2. **Figure 5** shows example series of keyframes at various values of r .

The pixel distance between keyframes was converted to the movement of fruit in the camera coordinate system to further calculate the interval between keyframes in the video. The theoretical interval of keyframes, $i_{t}\;,\;$ could be calculated by Equation 4.


(4) $$i_{t}=\frac{d_{p}\times d\times fps}{f_{x}\times v}\;\;\;\;\;\;$$

where fps is the frame rate of the video. $f_{x}$ denotes the intrinsic parameters of the RealSense camera, v represents the traveling speed of MRP, and d stands for the average distance between the camera and the fruit. Equation 5 was used to calculate the nearest integer of $i_{t}$ to obtain the actual interval of keyframes, i .


(5) $$i=int\left(i_{t}\right)=int\left(\frac{w\times d\times fps}{f_{x}\times v\times r}\right)\;\;\;\;\;$$

where the variable d was assumed to be a constant in this study. i is only related to values of v and r , where i=g(v×r) . The counting-from-video problem was transformed into the statistics of fruit detection results of keyframes.

##### 3.2.2.2 Postprocessing

**Figure 6:**
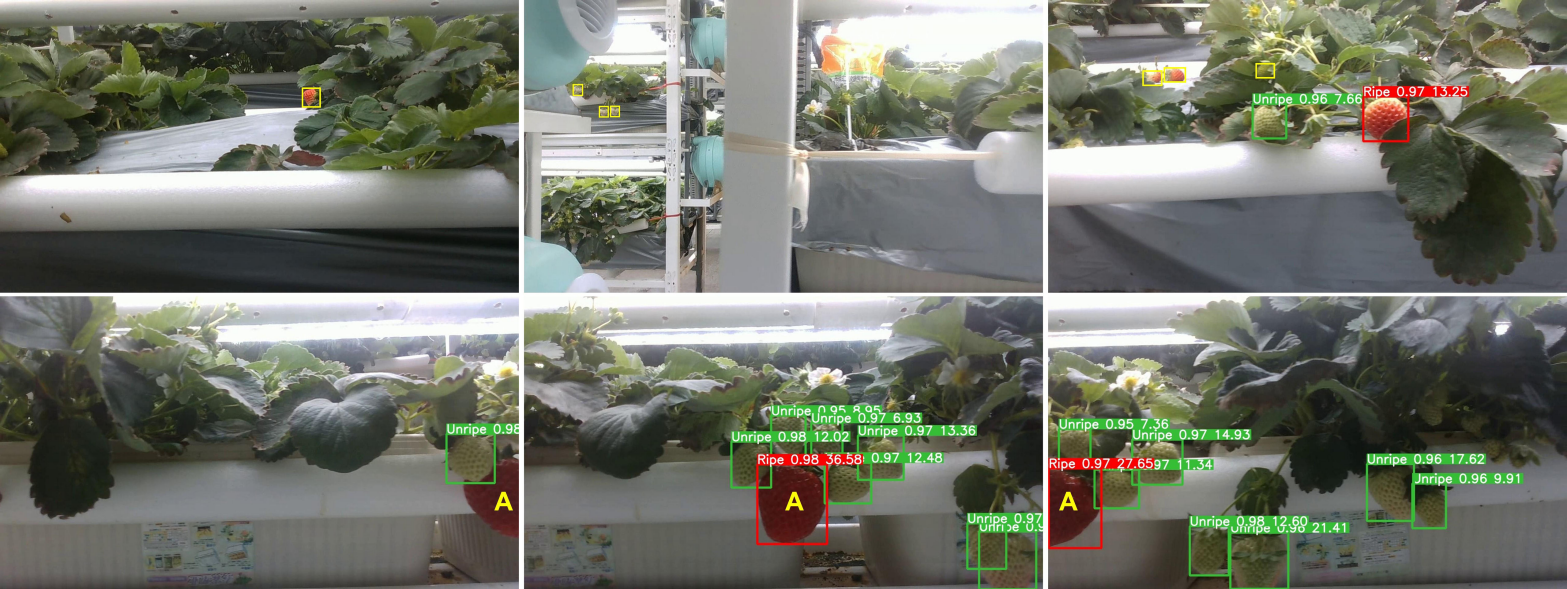
Upper row: Three example cases that needed to be processed by distance filtration. Strawberries annotated with yellow bboxes were not in the experimental areas and were not counted. Bottom row: Edge filtration was applied to process three consecutive keyframes ( r=2 ). The ripe fruit A was not counted since it was partly visible on the right edge of the left image. Fruit A was counted after it had moved to the left edge of the right image.

Postprocessing approaches were integrated to further improve the counting accuracy, including distance filtration, edge filtration, and multi-sequence average. Strawberries on other plant growing rows might enter the camera’s field of view during the MRP inspection process. The distance filtration approach based on the bounding box (bbox) size of the detection results was developed to eliminate the interference to counting by the strawberries located outside experimental areas. An edge filtration approach was used to prevent partially visible strawberries at the edge of the image from being counted repeatedly. Only the strawberries that appeared on the left edge were counted, and the strawberries that appeared on the right edge were ignored. **Figure 6** shows the two situations described above.

There existed errors in frame extraction between the actual interval of keyframes i and the theoretical interval of keyframes $i_{t}$ , $e=\left|\text{i}-i_{t}\right|$ . A multi-sequence averaging algorithm was developed to reduce the counting errors caused by the errors that occurred in the keyframe extraction process. The yield monitoring algorithm was presented as **Algorithm 1**:

Algorithm 1 Yield monitoring.
**Input:**  Threshold of keyframe interval i^s^, Threshold of errors of frame extraction e^s^, Threshold of the number of repetitive counting r^s^, MRP traveling speed *v*, Inspection video **V
Output:**  The number of ripe fruits in the video **V** , n
Initialize i^s^ =4, e^s^ =0.1, r^s^=15
Initialize 
$\text{R}=\;{\left\{r_{j}\right\}}_{j=2}^{r^{s}}=\left\{2,3,\ldots ,r^{s}\right\}$

**1** 
${\text{I}}_{t}:=\;{\left\{i_{t}j|i_{t}j:=g(r_{j}\times v),r_{j}\epsilon \text{R}\right\}}_{j:=2}^{r^{s}}$
 // Calculated by Eq. (4)**2** 
$\text{I}:={\left\{i_{j}|i_{j}:=\left(i_{tj}\right),i_{tj}\epsilon {\text{I}}_{t}\right\}}_{j:=2}^{r^{s}}$
 // Calculated by Eq. (5)**3** 
$E:={\left\{e_{j}|e_{j}:=\left|i_{tj}-i_{j}\right|,i_{tj}\epsilon I_{t},i_{j}\epsilon I\right\}}_{j:=2}^{r^{s}}$

**4** 
$R^{e}:={\left\{r_{j}|r_{j}\epsilon \text{R},e_{j}>e_{s},e_{j}\epsilon \text{E}\right\}}_{j:=2}^{r^{s}}$

**5** 
${\text{R}}^{i}\;:={\left\{r_{j}|r_{j}\epsilon \text{R},i_{j}<i_{s},i_{j}\epsilon \text{I}\right\}}_{j:=2}^{r^{s}}$

**6** 
${\text{R}}^{s}\;\text{R}-{\text{R}}^{e}\cap {\text{R}}^{i}$

**7** 
${\text{E}}^{s}:={\left\{e_{j}|e_{j}\epsilon E,r_{j}\epsilon \text{R}\right\}}_{j:=2}^{r^{s}}$

**8** 
${\tilde{E}}^{s}:=𝒮ort\left({\text{E}}^{s}\right)$
  // 𝒮ort: To sort E^s^ to get an ascending-order array 
${\tilde{E}}^{s}$

**9** **if** 
${\tilde{E}}^{s}\left[2\right]>e^{s}$

**then**         
$E^{c}:=\left\{{\tilde{E}}^{s}\left[0\right],{\tilde{E}}^{s}\left[1\right]\right\}$

**else**         

$E^{c}:=\left\{{\tilde{E}}^{s}\left[0\right],{\tilde{E}}^{s}\left[1\right],{\tilde{E}}^{s}\left[2\right]\right\}$

**10** 
${\text{R}}^{c}:={\left\{r_{j}|r_{j}\epsilon {\text{R}}^{s},e_{j}\epsilon E^{c}\right\}}_{j:=2}^{r^{s}}$

**11** 
$I^{c}\;:={\left\{i_{j}|i_{j}\epsilon I,r_{j}\epsilon R^{c}\right\}}_{j:=2}^{r^{s}}$
 // **R**
^c^: The group of filtered intervals of keyframes**12** 
$S\;:=\{s_{r_{j}}|s_{r_{j}}:=ℰ\left(V,i_{j}\right),r_{j}\epsilon R^{c},i_{j}\epsilon I^{c}\}$
 // ℰ: Toextract keyframes from V at interval i_j_
**13** 
$S^{F}:=\{s_{r}^{F}|s_{r}^{F}:=ℱ\left(s_{r}\right),s_{r}\epsilon S,\;r\epsilon R^{c}\}$
 // ℱ: To applydistance and edge filtration**14** 
$N\;:=\{n_{r}|n_{r}:=\frac{C\left(s_{r}\right)}{r},s_{r}\epsilon S^{F},r\epsilon R^{c}\}$
// 𝒞: To countthe ripe fruit in s_r_
**15** 
n := 𝒜verage(N)
 // 𝒜verage: To average allthe sequence results in **N**.

## 4 Procedure of experiments

**Figure 7:**
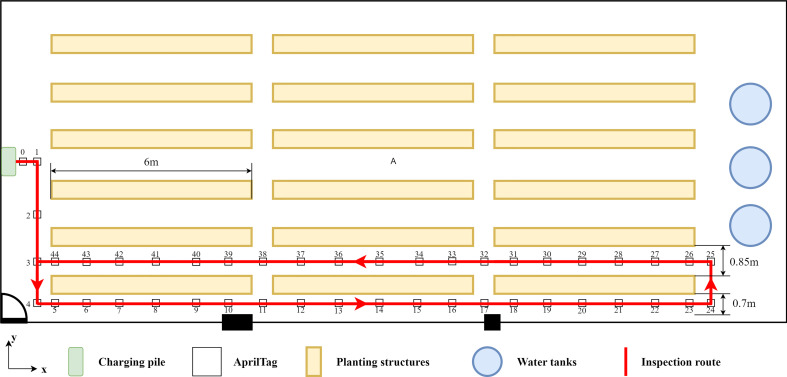
Schematic diagram of the experimental scene and inspection route.

In this study, experiments were carried out at a commercial strawberry plant factory (**Figure 2**) in December 2022. Fragaria × ananassa Duch. cv. Yuexin plants bred by the Zhejiang Academy of Agricultural Sciences (Hangzhou, Zhejiang, China) were cultivated on four-tier planting structures. The experiments were conducted on a row of three four-tier planting structures near a wall. There were 12 planting pots in every tier of each planting structure, and five strawberry plants were grown in each planting pot. Experiments were carried out on a total of 720 strawberry plants (i.e., 5 plants/pot × 12 pots/tier × 4 tiers/planting structure × 3 planting structures = 720 plants). **Figure 7** shows the floor layout of the research facility and the MRP inspection route.

### 4.1 Navigation capability

#### 4.1.1 Mapping

The typical configuration of a plant factory is a corridor environment with repetitive and narrow planting structures, which brings significant challenges to the LiDAR-based SLAM algorithm in mapping operations. LIO-SAM, one of the advanced LiDAR-based SLAM algorithms, was implemented on the MRP to compare and prove the advantages of the proposed mapping algorithm. LIO-SAM is a real-time, tightly coupled Lidar-Inertial odometry with high odometry accuracy and good mapping quality (Shan et al., 2020). In order to satisfy the use of the LIO-SAM algorithm, a VLP-16 3D LiDAR scanner (Velodyne Lidar, California, USA) and a WitMotion HWT905 nine-axis attitude and heading reference system (AHRS) sensor (WitMotion, Shenzhen, China) were integrated within the MRP. The collection of the mapping dataset was conducted using the same approach mentioned in *Section 3.1*. The data of 3D LiDAR and nine-axis IMU were used in the LIO-SAM algorithm for pose estimation. The data of the monocular camera, IMU, and wheel encoders were used in the mapping algorithm of the ATI navigation system developed in this research. All optimization processes were conducted offline for the two algorithms. Another experiment was conducted to compare the mapping performances of the ATI navigation system, without and with loop closing optimization, to show the impact of optimization in this research. Mapping trajectories were used to evaluate the mapping performances of the three approaches.

#### 4.1.2 Localization

This experiment aims to test the ability of MRP to move to a desired location as expected. PA was used to evaluate the navigation performance in this research. The coordinate system is shown in the lower left corner of **Figure 7**. The positive direction of the *X*-axis is consistent with the movement direction of the MRP when inspecting strawberry plants. In the autonomous navigation mode, three tags at different positions (Tags 8, 12, and 21) were selected for testing PA. The MRP started from Tag 5 and navigated to the target tag at the traveling speed of 0.4 m/s after entering the Tag ID. The current position of the tag in the image coordinate system was recorded to compare with the tag’s position in the map generated by the ATI navigation algorithm. The same operations were repeated five times for each tag. Euclidean distance between two positions was represented as distance deviation, err_d . err_x represents the deviation in the x direction, and err_y represents the deviation in the y direction. The root mean squared error (RMSE) of five trails per tag was computed by Equation 6, and the RMSE of 15 trails of three tags was computed as PA.


(6) $$RMSE=\sqrt{\frac{1}{k}\sum_{l=1}^{l}err\_d_{l}{\;}^{2}}$$

where k is the number of trails. $err\_d_{l}$ represents the err_d in trail l .

### 4.2 Fruit detection and counting

#### 4.2.1 Fruit detection

A total of 80 videos were captured along the plant growing rows by farmers at a normal walking pace using an Intel^®^ RealSense™ D435i depth camera and a smartphone, under various illumination conditions, different strawberry growth scenes, and various strawberry growth stages (from March to July 2021). The dataset consisted of 1,600 frames that were extracted out of every 10 frames from the videos, with the images without strawberries manually removed. All strawberry fruits in the period of veraison were annotated by growers. Of those, every fruit having an 80% or more red area on its surface was annotated as a ripe fruit (Hayashi et al., 2010). Other fruits were annotated as unripe ones. The dataset, including 2,327 ripe strawberries and 2,492 unripe strawberries, was randomly divided into train, validation, and test sets at the ratio of 8:1:1.

The strawberry ripeness detection model, YOLOv5l6, was implemented using the PyTorch framework. The modeling process was performed on a Linux workstation (Ubuntu 16.04 LTS) with two Intel Xeon E5-2683 Processors (2.1G/16 Core/40M), 128 GB of RAM, and four NVIDIA GeForce GTX 1080Ti graphics cards (11 GB of RAM). Taking a mini-batch size of 16, the SGD optimizer was adopted with a decay of 0.0001 and a momentum of 0.937. The best performance was achieved under the initial learning rate of 0.01. The number of warmup epochs and total training epochs were set to 3 and 90, respectively. The best model weight was chosen according to the value of mean average precision (mAP) (Everingham et al., 2010) calculated on the validation set. The chosen model was evaluated on the test set by mAP@0.5 (at the IoU threshold of 0.5).

#### 4.2.2 Fruit counting

False detections and missed detections of fruit in a particular frame cannot be corrected by any other frames. Therefore, in this study, a counting algorithm was developed to count every fruit multiple times (a predetermined number of times that is equal to or greater than 2) in order to improve the accuracy of the fruit counting. The performance of the proposed algorithm was affected by r , i , and e . As mentioned in *Section 3.2*, i and e were related to the value of r . In this experiment, various values of r were tested to build the fruit counting algorithm with a robust performance. The MRP traveled at the speed of 0.3 m/s along the aisle between plant growing rows to capture the phenotypic data of each plant in the experimental region. Both video data captured by the RealSense camera at the actual frame rate of 29.72 fps and data from navigation sensors were recorded in the rosbag format at a unified timestamp. The MRP inspected and recorded all the data twice for each tier of plant growing rows. A total of eight videos were collected in this experiment. Fruit detection was performed on the eight videos. The number of ripe strawberry fruit in the results produced by the detection algorithm, $n_{GT}^{C}$ , was manually counted as the ground truth of the fruit counting algorithm to exclude the impact of the fruit detection algorithm and evaluate the performance of the fruit counting algorithm alone. The yield monitoring algorithm results, n , were then estimated using the proposed algorithm without multi-sequence averaging (one of the three postprocessing techniques mentioned in *Section 3.2.2*). The thresholds $e^{s}$ and $i^{s}$ , mentioned in Algorithm 1, can be determined by selecting a number of smaller relative error rates of fruit counting, $err^{C},$ calculated by Equation 7.


(7) $$err^{C}=\frac{\left|n-n_{GT}^{C}\right|}{n_{GT}^{C}}\times 100\%\;\;\;\;$$

### 4.3 Inspection capability

In this experiment, the inspection capability of MRP was tested at various traveling speeds of 0.2, 0.3, and 0.4 m/s. The inspection capability was a system performance that included mobility for multiple-location data acquisition and monitoring of strawberry yield.

#### 4.3.1 Motion control

The experiment in this study was conducted three times to test the motion control performance of MRP at three different traveling speeds. In the navigation mode, MRP was programmed to start from the first tag (Tag 5) and stop at the last tag (Tag 23) position in the aisle. The distance error, linear velocity, yaw error, and angular velocity of the MRP were recorded in the rosbag format with a frame rate of 50 Hz as the errors and outputs of the control system. Motion stability and angular tracking accuracy were considered to evaluate the effectiveness of the proposed method.

#### 4.3.2 Yield monitoring

The accuracy of the yield monitoring algorithm is a system performance to evaluate both fruit detection and counting processes. The variables  r , i , and e corresponding to three different traveling speeds could be calculated by repeating the operations mentioned in *Section 4.2.2* in the same experimental area on different dates. This experiment was conducted three times to test the accuracy of the yield monitoring algorithm at three traveling speeds of MRP. For each experiment, MRP inspected and recorded all the data twice for one of the four tiers of the plant growing rows. A total of 24 videos were collected in this experiment. The number of ripe strawberries in the raw video, $n_{GT}^{Y}$ , was determined by growers as the ground truth of the yield. The relative error rate of yield monitoring, $err^{Y}$ , could be calculated by Equation 8.


(8) $$err^{Y}=\frac{\left|n-n_{GT}^{Y}\right|}{n_{GT}^{Y}}\times 100\%\;\;\;\;$$

## 5 Results and discussion

### 5.1 Navigation capability

#### 5.1.1 Mapping

**Figure 8:**
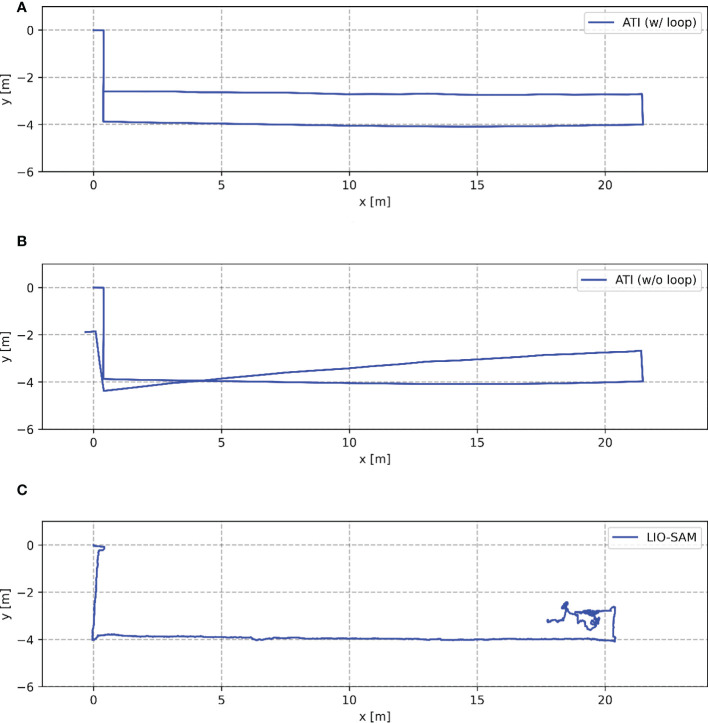
Comparison of trajectories obtained by the three mapping approaches running in the experimental area. **(A)** shows the mapping trajectory processed by the ATI mapping approach with the loop closing optimization. **(B)** shows the mapping trajectory processed by the ATI mapping approach without the loop closing optimization. **(C)** shows the mapping trajectory processed by the LIO-SAM algorithm with optimized parameters.

As shown in **Figure 8**, two continuous and smooth trajectories were obtained using our ATI mapping approach (a and b). The two trajectories almost coincided before Tag 27. The trajectory in **Figure 8B** was the non-optimized result, which the MRP was not able to return to the charging pile (origin) due to cumulative errors of the system. **Figure 8A** shows the mapping trajectory processed by the ATI mapping approach with the loop closing optimization that was accomplished by making the path defined by Tags 0, 1, 2, and 3 the beginning segment and the path defined by Tags 3, 2, 1, and 0 the ending segment of the trajectory. The beginning tags (numbers 0, 1, 2, and 3) were detected in a reversed order when MRP was on the way back to the starting point, Tag 0. The global PGO was successfully performed to eliminate the cumulative errors and obtain a consistent and undistorted trajectory during the mapping process. The mapping trajectory coincided with the AprilTags pasted on the ground in the experimental area (**Figure 7**).

In contrast, a jittery mapping trajectory was obtained by LIO-SAM under the same movement of MRP (**Figure 8C**). Degeneracy occurred when MRP traveled back and turned to a new long aisle, i.e., starting from the position of Tag 25 in **Figure 7**. The estimated odometry oscillated around the same position. It is worth mentioning that the mapping results presented in **Figure 8C** were obtained by the LIO-SAM algorithm with optimized parameters. The original LIO-SAM failed at the second turn of the inspection route, i.e., starting from the position of Tag 4 in **Figure 7**. The experimental results show that the LiDAR-based SLAM algorithm failed in the environment of the plant factory. Our ATI navigation algorithm was effective and robust in the mapping process.

#### 5.1.2 Localization

In the PA experiment, Tags 8, 12, and 21 were selected as target positions (**Figure 7**). Tests were repeated five times for each tag. The range of RMSE of each tag was found to be between 8.6 and 14.8 mm (**Table 1**). The overall RMSE of PA was 13.0 mm. Each tag could be effectively observed using the proposed ATI navigation algorithm, which showed the robustness of the positioning system. The positioning results of the algorithm in the x and y directions are all biased to the same side (**Tables 2**, **3**). The external parameters among the wheel encoders, IMU, and monocular camera were estimated from the mechanical drawings with no calibration process in this research. The PA of the system could be further improved by automatic and accurate calibration of the navigation sensors and the optimization of fusion of wheel encoders and IMU.

**Table 1:** Positioning accuracy of the ATI navigation algorithm.

Tag ID	*err_d* (mm)						RMSE (Tag)	RMSE (All)
	1	2	3	4	5	Avg		
8	9.6	10.5	8.5	7.2	6.9	8.5	8.6	13.0
12	17.2	16.8	12.6	12.5	14.0	14.6	14.8	
21	13.1	16.6	17.1	16.6	6.6	14.0	14.5	

err_d , distance deviation is the Euclidean distance between the current position of the tag in the image coordinate system and the position of the tag in the map generated by the ATI navigation algorithm.

**Table 2:** Positioning accuracy in the *x* direction of the ATI navigation algorithm.

Tag ID	*err_x*(mm)						RMSE (Tag)	PA (*x*)
	1	2	3	4	5	Avg		
8	−9.1	−10.4	−8.3	−5.9	−5.1	−7.8	8.0	11.2
12	−16.5	−14.4	−10.1	−11.1	−11.5	−12.7	12.9	
21	−8.6	−15.2	−16.2	−11.7	5.2	−9.3	12.1	

**Table 3:** Positioning accuracy in the *y* direction of the ATI navigation algorithm.

Tag ID	*err_y* (mm)						RMSE (Tag)	PA (*y*)
	1	2	3	4	5	Avg		
8	−3.2	−0.8	−2.0	−4.1	−4.6	−2.9	3.2	6.5
12	−4.7	−8.7	−7.6	−5.8	−8.0	−7.0	7.1	
21	−9.9	−6.7	−5.4	−11.8	−4.0	−7.6	8.1	

### 5.2 Fruit counting capability

The best model weight was chosen according to the mAP@0.5 value of 0.994 for ripe strawberries calculated on the validation set. We have found that an mAP@0.5 value of 0.945 could be obtained on the test set. Strawberry growth scenes with occlusions could be identified accurately by the fruit detection model.

We have found that there was little change in $i_{t}$ and i when the value of r was more than 15 and the value of v was 0.2, 0.3, or 0.4 m/s. The value of r was set from 2 to 15, and the value of v was 0.3 m/s in this experiment. The corresponding i and e values and the relative error rate of fruit counting, $err^{C}$ , were computed and are shown in **Table 4** in ascending order according to e values. The value of $err^{C}$ generally increased as the increase of e . When the value of e was more than 0.1, the $err^{C}$ was relatively large and fluctuated. When the value of i was relatively small, the impact of e on $err^{C}$ was more obvious. The value of $i^{s}$ as set as 4 through the observation of the experimental results. In this experiment, the values of r were chosen as 15, 10, and 6. The final $err^{C}$ was computed as 3.3%. There also existed several limitations. We assumed that the value of d was constant. However, the variance in the distance between strawberries and the RealSense camera existed in the production scene, which affected the accuracy of the algorithm. The problem could be addressed by dynamically introducing accurate values of d captured by the depth camera into the algorithm. When v is high, the overlaps of two neighboring frames will be fewer. This will, in turn, limit the range of r values and the tolerable error rate will become smaller.

**Table 4:** The relative error rate of fruit counting under different algorithm setups.

Setup				Counting results of various videos								*Avg err^c^*
*r*	*i_t_*	*i*	*e*	1_1	1_2	2_1	2_2	3_1	3_2	4_1	4_2	
15	2.029	2	0.029	28	29	31	31	43	46	37	36	0.032
10	3.044	3	0.044	28	29	31	31	43	46	37	36	0.032
6	5.073	5	0.073	29	29	30	31	43	46	37	35	0.035
5	6.088	6	0.088	28	29	31	31	43	47	37	35	0.038
3	10.146	10	0.146	30	26	29	30	44	46	36	35	0.052
14	2.174	2	0.174	30	31	33	34	46	49	40	38	0.049
8	3.805	4	0.195	27	27	30	30	41	42	35	34	0.072
2	15.219	15	0.219	28	29	31	29	44	49	36	33	0.059
11	2.767	3	0.233	25	26	28	28	39	42	34	32	0.116
13	2.341	2	0.341	33	33	36	36	50	53	43	41	0.133
7	4.348	4	0.348	31	31	34	34	46	48	40	39	0.058
9	3.382	3	0.382	31	32	34	34	48	51	41	40	0.083
4	7.610	8	0.390	27	27	30	30	41	42	35	34	0.072
12	2.537	3	0.4635	23	24	26	26	36	38	31	30	0.185
$n_{GT}^{C}$				30	30	32	32	44	44	37	37	

1_1 and 1_2 are the first and second videos of strawberries grown on the first tier, respectively. $Avg\;err^{C}$ is the average relative error rate of fruit counting, $n_{GT}^{C}$ is the number of ripe strawberry fruit in the detection results.

### 5.3 Inspection capability

#### 5.3.1 Motion control

**Figure 9:**
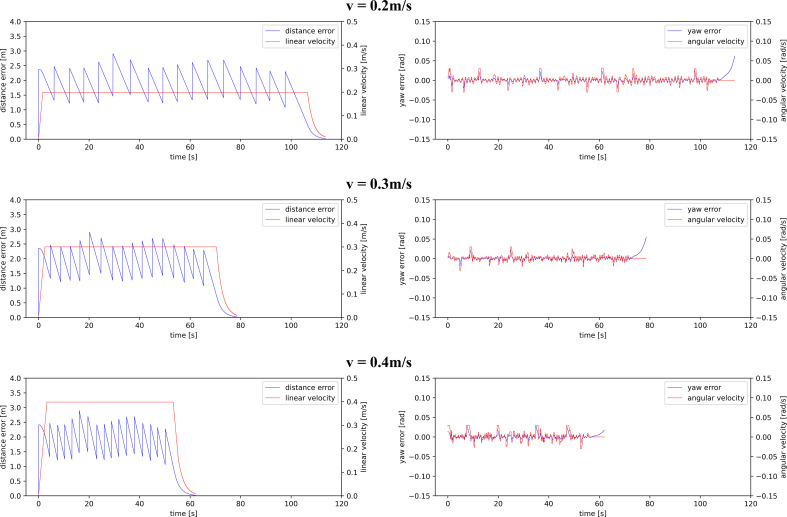
The performance of distance and heading controller at various MRP speeds.

The motion control system worked stably at the nominal MRP traveling speeds of 0.2, 0.3, and 0.4 m/s. The performance of the distance controller and heading controller at various speeds is shown in **Figure 9**. The inspection durations at the three set speeds are 113.6, 78.6, and 62.1 s, respectively. The overall average speeds are 0.189, 0.273, and 0.346 m/s, respectively.

On the left of the figure, the blue lines represented the distance between MRP and the target position in the local path planner (*Section 3.1*), $Dis_{local}$ , during the navigation process. At the start, the value of $Dis_{local}$ was approximately 2.4 m, which was the distance between Tag 5 and Tag 7. As the robot moved forward, the value of $Dis_{local}$ decreased linearly. When the MRP reached Tag 6, the local target was updated to Tag 8. At this time, the value of $Dis_{local}$ returned to approximately 2.4 m, which was the distance between Tag 6 and Tag 8. When the MRP reached Tag 22, the local target was no longer updated. The value of $Dis_{local}$ faded to zero as the robot moved towards the global target, Tag 23. MRP accelerated from zero to a set traveling speed, maintained the speed during the inspection, and gradually decelerated until reaching the global target, Tag 23, without an overshoot. On the right of the figure, the red lines represented the heading from MRP to the target position in the local path planner, $Yaw_{local}$ , during the navigation process. The value of $Yaw_{local}$ was within 0.01 rad most of the time and occasionally rose to 0.03 rad due to the updates of the target positions in the local path planner, which had little effect on the phenotypic data acquisition. The control system ensured smooth and low-error motions at various traveling speeds of MRP for stable quality of video collection.

#### 5.3.2 Yield monitoring

The $err^{C}$ and $err^{Y}$ of 24 test videos (8 videos per MRP traveling speed) were calculated and shown in **Table 5**. We found that the system showed robust monitoring results at various MRP traveling speeds, of which $err^{C}$ was between 2% and 3%, and $err^{Y}$ was between 6% and 10%. The best yield estimation performance was found to have an error rate of 6.26% at the MRP traveling speed of 0.2 m/s. The four ties of plant growing row in the experimental area corresponded to the four strawberry growth densities. Our algorithm had high robustness when dealing with scenes with various fruit densities.

**Table 5:** Yield monitoring performance comparison at various speeds of MRP.

Setup				Video ID	*n*				*Avg err^c^*	*Avg err^Y^*
*v* (m/s)	*r*	*i*	*e*		T1	T2	T3	T4		
0.2	15	3	0.044	1	34	52	87	70	0.0265	0.0626
	9	5	0.073	2	35	55	88	69		
0.3	15	2	0.029	1	37	54	88	71	0.0229	0.0905
	10	3	0.044	2	36	53	90	72		
	6	5	0.073							
0.4	11	2	0.075	1	37	51	85	71	0.0252	0.0711
	6	4	0.195	2	38	52	84	70		
$n_{GT}^{C}$				1	36	54	85	70		
$n_{GT}^{Y}$				2	32	51	83	65		

T1 is the first tier of the plant growing row in the experimental area. n is the result of the yield monitoring algorithm. $n_{GT}^{C}$ is the number of ripe strawberry fruit in the detection results. $n_{GT}^{Y}$ is the number of ripe strawberries in the raw video. $err^{C}$ is the relative error rate of fruit counting. $\;err^{Y}$ is the relative error rate of yield monitoring.

The same strawberry appeared differently in various frames due to the changes in shooting angles during the movement of MRP. An unripe strawberry might be detected as a ripe or unripe one from various angles due to the distribution of red color on the fruit, which made $n_{GT}^{Y}$ smaller than $n_{GT}^{C}$ . The proposed yield monitoring approach is a detection-based pipeline, in which false detections caused the higher $err^{Y}$ . In order to meet the above challenges and obtain higher yield estimation accuracy, there exists a potential solution, which is to process with the original video data. Videos captured by the MRP could provide both spatial and temporal information for better tracking and detecting a single fruit. However, a large amount of needed computational time was the limitation of this solution.

## 6 Conclusion

In this study, we have developed software and hardware of an MRP, consisting of an AMR and an MPR, which can capture temporal–spatial phenotypic data within the whole strawberry factory. This paper reported two basic capabilities of the MRP, navigation for multiple-location data acquisition and strawberry yield monitoring. An ATI navigation algorithm was developed to address the challenges of accurate navigation within the repetitive and narrow structural environments of a plant factory. The MRP performed robustly at various traveling speeds tested with a PA of 13.0 mm. A counting-from-video yield monitoring method that incorporated keyframes extraction, fruit detection, and postprocessing technologies was presented to process the video data captured by MRP’s inspection for production management and harvesting schedules. The yield monitoring performance was found to have an error rate of 6.26% when the plants were inspected at a constant MRP traveling speed of 0.2 m/s. The temporal–spatial phenotypic data within the whole strawberry factory captured by the MRP could be further used to dynamically understand plant growth and provide data support for growth model construction and production management. The MRP’s functions are expected to be transferable and expandable to other crop production monitoring and cultural tasks.

## Data availability statement

The raw data supporting the conclusions of this article will be made available by the authors, without undue reservation.

## Author contributions

GR, TL, YY, and KT: conception and design of the research; GR and HW: Hardware design; GR, HW, and AB: data preprocessing, model generation and testing, visualization, and writing—original draft; GR, TL, YY, and KT: writing—review and editing. GR, HW, and AB: validation. TL, YY, and KT: Supervision. All authors contributed to the article and approved the submitted version.
